# Epigenetic Regulation of Epidermal Differentiation

**DOI:** 10.3390/epigenomes5010001

**Published:** 2021-01-01

**Authors:** Wiesława Leśniak

**Affiliations:** Laboratory of Calcium Binding Proteins, Nencki Institute of Experimental Biology of the Polish Academy of Sciences, 3 Pasteur Street, 02-093 Warsaw, Poland; w.lesniak@nencki.edu.pl; Tel.: +48-22-5892-327; Fax: +48-22-822-53-42

**Keywords:** epigenetic mechanisms, epidermal differentiation, DNA methylation, histone modifications, microRNAs, skin aging, skin diseases, wound healing

## Abstract

The epidermis is the outer part of the skin that protects the organism from dehydration and shields from external insults. Epidermal cells, called keratinocytes, undergo a series of morphological and metabolic changes that allow them to establish the biochemical and structural elements of an effective epidermal barrier. This process, known as epidermal differentiation, is critical for the maintenance of the epidermis under physiological conditions and also under stress or in various skin pathologies. Epidermal differentiation relies on a highly coordinated program of gene expression. Epigenetic mechanisms, which commonly include DNA methylation, covalent histone modifications, and microRNA (miRNA) activity, modulate various stages of gene expression by altering chromatin accessibility and mRNA stability. Their involvement in epidermal differentiation is a matter of intensive studies, and the results obtained thus far show a complex network of epigenetic factors, acting together with transcriptional regulators, to maintain epidermal homeostasis and counteract adverse effects of environmental stressors.

## 1. Introduction

**Figure 1:**
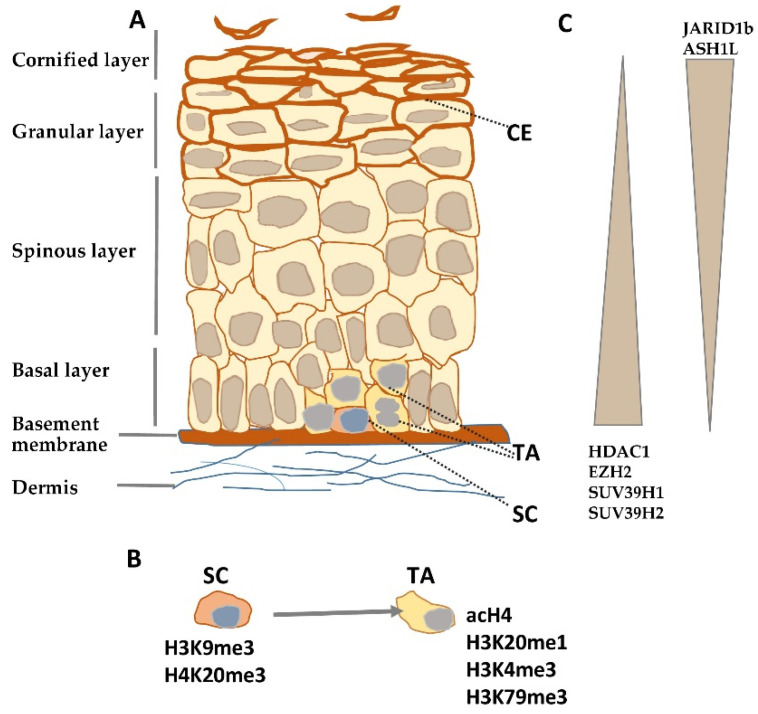
Histone modifying enzymes and histone modifications in epidermal differentiation. (**A**) Schematic representation of human epidermis. (**B**) Histone modifications typical for epidermal stem (SC) and transient amplifying (TA) cells. (**C**) Gradient of histone modifying enzyme expression in epidermis. CE—cornified envelope.

The epidermis constitutes the surface of the skin and is built of specialized epithelial cells called keratinocytes that are organized in several layers: Basal, spinous, granular, and the outermost—corneal layer [1]. Keratinocytes originate from two pools of quiescent epidermal stem cells (qESC), one residing in the basal layer of the interfollicular epidermis (IFE) and another, in a so-called “bulge”, situated in the hair follicle below the opening of the sebaceous gland; the bulge stem cells generate keratinocytes of the hair follicle (HF) lineage. Quiescent epidermal stem cells give rise to transient amplifying (TA) cells, which, in turn, generate “mature” keratinocytes that populate the basal epidermal layer or the outer root sheath of the hair follicle [2]. After an initial round of cell divisions, keratinocytes in the basal layer are pushed upward by newly generated cells, lose contact with the basal membrane, cease to proliferate and enter the differentiation phase. During differentiation, keratinocytes execute a highly ordered and time-synchronized synthesis of specific lipids and proteins necessary to build the cornified envelope, that is, a specialized sub-membranous structure, which provides a tight and resilient barrier against environmental hazards. This process is coupled with changes in keratinocyte morphology, i.e., the cells flatten, enucleate, and, when they reach the uppermost corneal layer, successively desquamate [1]. Thus, within about the 30 days necessary to complete this cycle in humans, keratinocytes pass through quiescence, transient proliferation stage, differentiation phase, and, finally, cell death (Figure 1A). Epidermal growth and differentiation entail a well-coordinated expression of multiple genes, which is orchestrated by various mechanisms of transcriptional and post-transcriptional control. This strict control is often challenged by external stressors (e.g., UV light, wounding), modulated by physiological factors (e.g., nutrient supply, aging), or dysregulated in skin diseases.

Genetic information, common to all individuals of a given species, is processed and modified by epigenetic mechanisms with the result that each individual cell or tissue acquires a different phenotype [3]. Epigenetic factors regulate gene expression at both the transcriptional and post-transcriptional levels and are especially important in developmental processes and cellular differentiation. DNA methylation/demethylation and histone modifications (e.g., methylation, acetylation, phosphorylation) alter chromatin structure and accessibility to the transcriptional machinery by facilitating or impeding the binding of chromatin-modifying complexes. On the other hand, microRNAs (miRNAs) induce cleavage of mRNAs or interfere with their translation. The extent of the contribution of epigenetic mechanisms to the control over epidermal differentiation is a subject of intensive studies. This review article offers an insight into how epigenetic factors contribute to and regulate keratinocyte differentiation.

## 2. Epigenetic Mechanisms Involved in Epidermal Differentiation

### 2.1. Histone Modifications

Histones are basic proteins that bind to DNA and organize the chromatin. Dimers of four core histones (H2A, H2B, H3, and H4) form an octamer, which, together with a 146 bp long fragment of DNA strand, constitutes the elementary chromatin unit, i.e., the nucleosome. In contrast, histone H1 binds to the internucleosomal (linker) DNA, stabilizing, and compacting the nucleosome structure [4]. The N-terminal histone tails, rich in basic amino acid residues, are subject to many covalent modifications, most of which (e.g., acetylation, methylation, phosphorylation, or ubiquitination) are reversible. These modifications, often referred to as a histone code, are catalyzed by specific groups of enzymes that include histone acetyltransferases (HATs), deacetylases (HDACs), methyltransferases (HMTs), and demethylases (HDMs). HATs and HDACs usually have broad specificity as to the histone type and amino acid position while HMTs and HDMs are more specific and usually methylate/demethylate a particular histone at a particular position and to a particular extent (e.g., mono-, di- or trimethylation of a lysine residue) [5,6]. Certain histone modifications, such as H3K4me3 H3K79me2/3, and H3K36me3 are found on promoters or gene bodies of actively transcribed genes and are often referred to as activating histone marks. Acetylation of lysine residues also promotes transcription since it neutralizes their positive charge and disrupts electrostatic interactions between histones and DNA, which results in less effective local DNA packaging [5]. On the other hand, H3K9me2/3, H3K27me2/3 and H4K20me3 and histone deacetylation are repressive histone modifications found on promoters of transcriptionally inactive genes. The activating or repressive effect on transcription of these or other histone modifications is exerted via a chain of protein interactions that ultimately engage ATP-dependent chromatin remodeling complexes. More specifically, modified histone residues serve as recognition sites for non-histone proteins endowed with specific modules e.g., so-called bromodomains, that can bind to various acetylated lysine residues, or chromodomains, that recognize particular methylated lysine residues with high specificity. These domains are present either in proteins (e.g., HATs) that are components of larger, chromatin-remodeling complexes containing, as a catalytic component, ATP-dependent RNA/DNA helicases, or in proteins, such as Brg1 [7] or the SWI/SNF family remodelers, that are RNA/DNA helicases themselves [8]. There are 4 families of chromatin-remodeling complexes with slightly different functionalities; all of them rely on ATP-derived energy to slide or remove the nucleosome to expose cis-elements important for transcription and/or to modulate the arrangement and stability of nucleosomes towards either higher chromatin compaction or relaxation [8].

Histone modifications constitute a crucial factor in skin development and morphogenesis as it has been documented by studies on embryonic ablation of histone-modifying enzymes such as deacetylases [9] or methyltransferases [10]. Numerous studies certify that they also shape the architecture and functionality of the adult epidermis by controlling epidermal growth and differentiation. Quiescent stem cells residing in the interfollicular epidermis possess a characteristic pattern of histone modifications. Namely, they were found to be enriched in inhibitory histone marks, H3K9me3 and H4K20me3, and to have a low level of activating modifications, such as acetylated histone H4 (acH4) and histone H4 monomethylated on lysine 20 (H4K20me1) (Figure 1B) [11]. Upon overexpression of c-Myc, a transcription factor that, under physiological conditions, induces the transition from quiescent to TA cells [12], the level of H3K9me3 was diminished while that of acH4 was increased, and there was, albeit transient, an increase in the H4K20me1 level [11]. A rise in the level of activating histone modifications, H3K4me3 and H3K79me3, was also observed at this stage in the hair follicle stem cells [13].

Results of experiments performed on mice with conditional knockout/knockdown or overexpression of histone-modifying enzymes in basal, keratin-14 (K14) expressing keratinocytes, and those obtained using cultured keratinocytes with altered expression of these enzymes, largely substantiate the observed changes in histone modification pattern occurring at the early stage of epidermal growth. For example, the loss of the Setd8 methyltransferase, which monomethylates H3K20, resulted in a lower level of this activating histone mark and led to inhibition of progenitor cell proliferation in the basal layer, which, in consequence, impaired epidermal differentiation [10]. *Setd8* deletion coincided with a diminished level of p63, a transcription factor that controls the balance between keratinocyte proliferation and differentiation by maintaining the proliferative potential of epidermal stem cells [14]. Knockout of *Ezh2*, encoding H3K27 methyltransferase, in the basal cells of neonatal mouse epidermis resulted in a lower proliferation rate and higher expression of epidermal differentiation markers, such as loricrin and filaggrin, synthesized by differentiating keratinocytes [15]. In this case, reduced proliferation of basal keratinocytes could be largely attributed to the de-repression of genes encoding cell cycle inhibitors, including p16^INK4a^. It was also found that promoters of many differentiation-specific genes bear the H3K27me3 inhibitory histone mark in undifferentiated keratinocytes and that the premature differentiation was caused by loss of this mark due to *Ezh2* knockout [15]. Interestingly, the effect of *Ezh2* knockout was very similar to that of Jmjd3, a H3K27 demethylase, overexpression, which provoked induction of genes encoding differentiation markers, such as keratin 1, keratin 10, S100A8, and involucrin, in primary human keratinocytes [16]. Conversely, siRNA-induced silencing of *Jmjd3* expression blocked the induction of differentiation genes that were marked by the presence of H3K27me3 on their promoters [16]. Knockout of *Suv39h1*, a gene encoding histone methyltransferase that imposes another inhibitory histone mark, H3K9me3, in HaCaT cells, a stable keratinocyte-derived cell line, also resulted in induction of differentiation-associated genes encoding keratin 10, desmoglein 1, S100A8, and late cornified envelope (LCE1) proteins [17]. In addition, a naturally occurring mutation in *Suv39h2* that results in enzyme inactivity provoked higher expression of genes encoding differentiation markers [18]. 

Depletion of histone deacetylases, Hdac1 and Hdac2, in the basal layer of mouse epidermis resulted in increased histone acetylation and enhanced keratinocyte proliferation leading to hyperplasia and thicker epidermis, although it also caused apoptosis and hair follicle dystrophy [19]. Silencing of Jarid1b, an enzyme, which demethylates H3 on lysine K4, reduced the level of the activating histone mark, H3K4me3, and led to diminished expression of differentiation markers in HaCaT cells, while overexpression enhanced differentiation and inhibited proliferation [20]. Studies on mice bearing a mutation that decreases transcription and expression of *Ash1l*, the gene that encodes an H3K36 methyltransferase, showed that adult animals had hyperproliferative epidermis with a more diffused localization of keratin 1 and keratin 14; the latter one, a marker of undifferentiated keratinocytes, being also present in the suprabasal layer [21]. The effect of knockout/down or overexpression of histone-modifying enzymes on epidermal growth/differentiation is summarized in Table S1, and changes in their expression during epidermal differentiation are illustrated in Figure 1C.

The above studies show that the control of epidermal differentiation by histone modifications is exerted over at least two important stages. Namely, histone modifications regulate the switch between epidermal stem cell quiescence and proliferation, as indicated by Sen and coworkers [16], but also the expression of epidermal differentiation genes. In the first case, the control is maintained over cell cycle inhibitor genes and/or transcription factors and pathways that supervise the transition between quiescence and proliferation. In the second case, inhibitory histone modifications are directly imposed on promoters of differentiation-induced genes in undifferentiated keratinocytes. Interestingly, as noted by several reports, the depletion of a particular histone-modifying enzyme did not cause a general induction or inhibition of differentiation-associated genes, which suggests that various histone modifications control the expression of individual genes or group of genes but not the whole differentiation program [15,16,17].

### 2.2. DNA Methylation

DNA methylation (addition of a methyl group to C-5 position of the cytosine ring in CpG dinucleotides) is another process essential for proper epidermal differentiation. DNA methyltransferase 1 (Dnmt1), is abundantly expressed in embryonic mouse epidermis [22] but, after birth, Dnmt1-positive staining becomes limited to the basal epidermal layer containing proliferating keratinocytes, which parallels Dnmt1 localization observed in adult human and mouse epidermis [22,23]. *Dnmt1* knockdown in primary human keratinocytes led to the loss of progenitor cells and premature differentiation in epidermal xenografts, which coincided with higher expression of Cdk inhibitors, e.g., p16^INK4a^ [23]. These findings were largely reproduced in mice, in which epidermal deletion of *Dnmt1* caused multiple alterations in hair growth associated mainly with reduced proliferation of TA cells [22]. The involvement of DNA methylation in this decisive step determining the fate of keratinocyte precursors is substantiated by results showing differences in DNA methylation, at single CpG resolution, between qHF-SC isolated in the telogen phase of hair growth, when stem cells are quiescent, and those isolated in anagen, when stem cells are activated to proliferate [24]. Furthermore, *Dnmt1* knockdown in keratinocytes resulted in higher expression of differentiation genes [23]. These findings led to the conclusion that DNA methylation by DNMT1 sustains progenitor proliferation by blocking the expression of cell cycle inhibitors and represses keratinocyte differentiation [23].

Changes in DNA methylation occurring during keratinocyte differentiation have been evaluated by several studies. Analysis of DNA methylation in undifferentiated and differentiated primary human keratinocytes, performed using MeDIP microarrays encompassing promoter sequences of nearly 25,000 genes, showed significant demethylation in 232 differentiation-induced gene promoters [23]. This analysis, which pointed to a substantial influence of DNA methylation/demethylation on the process of epidermal differentiation, has been recently challenged by a study analyzing changes in methylation of 850,000 CpGs during differentiation of primary human keratinocytes [25]. Results of the latter analysis revealed a stable methylation landscape and a lack of correlation between the methylation status and gene expression level. This finding was in agreement with earlier studies focused on genes of the Epidermal Differentiation Complex (EDC), which maintained their methylation status throughout the differentiation of primary keratinocytes despite large changes in expression [26,27]. This may indicate that the impact of DNA methylation on epidermal differentiation is exerted at the early stage, probably deciding on the balance between proliferation/quiescence of progenitor cells, with a lesser effect on gene expression in keratinocytes already poised for differentiation.

### 2.3. MicroRNAs

MiRNA expression in the skin and differences in the level of particular miRNAs at different stages of epidermal biogenesis, starting from the embryonic stage, were described soon after recognition of the role of miRNAs in gene expression regulation in higher organisms [28]. The essential role of miRNAs in skin biogenesis was revealed by studies on the consequences of epidermal-specific deletion of genes encoding miRNA processing enzymes *Dicer* and/or *Drosha* [28,29]. The resulting inhibition of miRNA generation had a profound effect on mouse skin morphogenesis, especially affecting the development of hair follicles, due to reduced proliferation of hair follicle keratinocytes and loss of progenitors, while the interfollicular epidermis largely preserved its morphology and proliferation potential [28]. In line with this finding, other studies provided evidence of differential expression of miRNAs in epidermal and hair follicle lineages [30], which may be indicative of differences in the epigenetic control in these two populations of epidermal progenitor cells, at least when miRNAs are concerned. The pronounced effect of *Dicer* knockout on hair follicle development correlated with the reduction in Wnt signaling, which is essential for hair follicle formation through securing proper communication between the dermis and epidermis [31]. Furthermore, the pattern of miRNAs expression in hair follicles was shown to be different in telogen and anagen, pointing to the role of miRNAs in hair cycle control [32,33].

Regarding keratinocyte differentiation, several studies documented the pattern of changes in miRNA expression during the course of human keratinocyte differentiation in vitro and in vivo [34,35,36]. There is a number of reports documenting, through both knockout/knockdown and overexpression of individual miRNAs, their role in epidermal development and differentiation. For example, the miR-184 knockout mouse model displayed epidermal hyperplasia, combined with increased p63 expression, while overexpression of miR-184 induced hypoplasia and enhanced Notch signaling, without any obvious effect on the hair follicle [37]. As indicated earlier, p63 is a transcription factor that maintains the proliferative potential of epidermal stem cells, while Notch signaling promotes keratinocyte differentiation [38]. Although miR-184 was shown not to target p63 directly, there are other miRNAs that regulate p63 level, among them miR-203, whose expression is inversely correlated with that of p63 along the path of keratinocyte differentiation [36,39]. Another miRNA, miR-214, was shown to target β-catenin and cause lower activity of the Wnt pathway [32]. Overexpression of miR-214 in a mouse model caused a reduction in epidermal thickness and lower keratinocyte proliferation rate as well as hair follicle loss. These examples suggest that miRNAs are involved in the control of epidermal fate at the critical point between proliferation and differentiation. The role of numerous other miRNAs in skin morphogenesis and epidermal differentiation has been described in some recent review articles [36,40].

## 3. Epigenetics in Psoriasis and Other Inflammatory Skin Diseases

Skin diseases markedly affect skin integrity and disturb epidermal differentiation. Epigenetic alterations have been commonly observed both in skin neoplasms and in chronic diseases with an inflammatory background, such as psoriasis or atopic dermatitis. The involvement of epigenetic factors in psoriasis, a chronic inflammatory disease characterized by hyperproliferation and incomplete differentiation/cornification of keratinocytes resulting in a thickened epidermis, has been intensively studied, and the published data give a good idea of their importance in this pathology. Analysis of psoriatic skin biopsies revealed dysregulation of multiple epigenetic mechanisms, including aberrant DNA methylation [41] and hydroxymethylation [42,43], alterations in histone modifications [44] and miRNA expression [45]. Interestingly, these differences could be observed not only between the skin of healthy individuals and psoriatic patients but also, although to a lesser extent, between patient’s uninvolved and lesional skin [45,46,47,48]. Such differences are indicative of early pathological changes occurring before the symptoms manifest themselves and may serve as markers of early stages of pathology.

Regarding DNA methylation, the number of differentially methylated sites (DMS) and/or regions (DMR) identified between samples derived from psoriatic skin and that of healthy donors varied depending on the method used and the total number of CpG analyzed [41,49], with the recent most extensive analysis of 2–4 million total CpGs in epidermal samples reporting tens of thousands DMS [46]. Differentially methylated CpGs were mostly located in introns [41] and enhancers [50], and there were fewer differences in CpG methylation in gene promoters. According to gene ontology, changes in CpG methylation were noted in genes classified mostly to categories such as cell cycle, apoptosis, immune system regulation, cell communication, signal transduction, which are processes severely impaired in psoriasis [41,49]. Although the overall correlation between methylation and expression of proximal genes was rather low [49,50], there were nonetheless a number of genes, including the one encoding p16^INK4a^, a cell cycle inhibitor [51], several psoriasis-associated genes [46], and some genes located in the EDC [49,52], for which methylation status could be correlated with expression level in psoriatic skin. Furthermore, hypomethylation of p16^INK4a^ correlated with increased psoriasis severity [51]. Importantly, changes in the methylation profile of psoriatic skin could be partly reversed after therapeutic treatment [49,50]. This, on the one hand, indicates that aberrant methylation may contribute to the pathology of psoriasis and, on the other hand, provides a strong argument that current therapies not only alleviate symptoms but act at the roots of the pathology.

Changes in miRNA expression have also been observed in psoriatic skin. For example, 42-upregulated and 5-downregulated miRNA species were identified in the skin of psoriasis patients in one study [53], while at least 250 differentially expressed miRNAs have been reported in the recent analysis [54] and 104 when isolated keratinocytes were studied [47]. A smaller number, that is 28 miRNAs, differentiated lesional from non-lesional skin in patients with psoriasis [45]. miRNA-mRNA correlation analysis indicated that these miRNAs were involved in regulating 56.6% of all mRNAs that were differentially expressed in psoriatic skin [45]. Most upregulated mRNAs belonged to the “immune response” category, while those downregulated were enriched in the “cell remodeling” category. Examples of the role of individual miRNA species in the context of regulating inflammatory cytokine expression, etc., can be found in [45,54] and other studies.

Evidence of altered histone modifications in psoriatic skin is also beginning to accumulate. For example, the H3K27me3 histone mark and Ezh2 level were found to be higher in psoriatic skin [44]. As described above and indicated in Table S1, *Ezh2* knockout in mouse epidermis inhibited cell proliferation, it is not surprising that increased Ezh2 level in lesional epidermis correlated with higher keratinocyte proliferation and could contribute to psoriatic hyperplasia. Pharmacological inhibition of EZH2 reduced both H3K27me3 level and cell proliferation [44]. Interestingly, the level of methylated H3K27 was different in psoriatic patients who responded or did not respond to therapeutic drug treatment [55]. Another study found a reduced level of the inhibitory histone mark, H3K9me2, in psoriatic skin that correlated with increased expression of IL-23, one of the interleukins that contribute to chronic inflammation in psoriasis [48].

Extensive changes in epigenetic mechanism have also been recognized in atopic dermatitis [56,57] and other inflammatory skin disorders [58,59]. Regarding DNA methylation, a study by Rodrigez et al. [60] found 127 DMSs (for about 27,000 analyzed) between control and lesional skin of atopic dermatitis patients. Methylation in several of these sites correlated with altered expression of S100A genes located in the EDC and genes encoding keratins. In another study analyzing 450,000 CpGs, differential DNA methylation was observed for 19 genes involved in a number of processes related to atopic dermatitis, including regulation of the immune response, activation of lymphocytes, cell proliferation, apoptosis, and differentiation of the epidermis [61]. As with psoriasis, less pronounced differences in DNA methylation were observed between non-lesional and lesional skin of patients suffering from atopic dermatitis. Aberrant DNA methylation was also observed within the filaggrin-encoding gene in people carrying its loss of function variants associated with the highest risk of eczema development [62].

## 4. An Insight into Epigenetic Mechanisms Involved in Skin Aging, Wound Healing, and Defense against Environmental Stressors

The notion that lifestyle and environmental factors can shape the organismal phenotype through inducing epigenetic alterations has been in circulation in the scientific milieu for quite a time before the first experimental evidence documenting changes in DNA methylation upon aging had been published [63]. Skin is in direct contact with the external environment and is, therefore, exposed to multiple environmental hazards, which may cause various types of injuries (mechanical, thermal, chemical, etc.) that can disturb epidermal differentiation. In addition, as an integral part of the organism, the skin is subject to natural life processes, such as aging. Presently, epigenetic factors are recognized players in processes that are activated in the skin/epidermis to protect from, counteract, or adapt to the adverse effects of external factors.

In regard to skin aging, methylation analysis of epidermal samples from a large cohort of young and old donors revealed altered methylation in about 13% of the examined sites; however, in the majority of cases, the changes were quantitatively very small [64]. In general, DNA was slightly more methylated in epidermal samples from older patients, especially in CpG islands. Another study, however, revealed more robust changes in DNA methylation with age, manifested by hypomethylation of large blocks of heterochromatin [65]. Nonetheless, in both studies, despite individual differences and the size of the effect, the pattern of age-related changes in DNA methylation in the epidermis appeared highly reproducible and strongly correlated with chronological age. As in other tissues, age-related changes in DNA methylation in the epidermis could not be unequivocally correlated with the level of gene expression and were thus interpreted as sustaining rather than altering the expression pattern [64,66].

A miRNA microarray screening of keratinocytes obtained from people in three age groups (infants, young adults, and aged adults) identified 60 miRNAs differentially expressed between at least two of these groups [67]. Differences were observed mainly between infants and both adult groups, suggesting that most miRNAs start to be up- or down-regulated upon transition between infancy and adulthood. In agreement with this observation, another study, comparing miRNA expression between young and aged adult sun-protected skin, failed to identify any significant differences in miRNA expression [68]. Overexpression of one of the miRNAs upregulated with age, miR-30a, resulted in impaired epidermal differentiation, manifested by lower expression of differentiation markers (keratin 1, keratin 10, involucrin) and the presence of keratin 14, the marker of undifferentiated keratinocytes, throughout all layers of epidermis reconstructed from primary human keratinocytes [67]. Functionally, such an epidermis had compromised barrier function and increased rate of keratinocyte apoptosis—two features of the aging epidermis. Accordingly, several potential targets of miR-30a were shown to be reduced in aged skin [67]. MiR-21 is another miRNA upregulated in aged mice and human skin. As in the case of miR-30a, overexpression of miR-21 resulted in downregulation of genes encoding keratin 1, keratin 10, keratin 14, and involucrin, and in dysregulated epidermal differentiation [69]. More information on skin/epidermis-specific miRNAs with altered expression with age can be found in a review article [70].

Skin exposure to UV light is a well-recognized factor contributing to skin cancer and skin aging. Changes in DNA methylation associated with UV irradiation have been summarized in the article by de Oliveira and colleagues in the special issue of Epigenomes devoted to Epigenetic Regulation of Epidermal Differentiation [71]. Besides DNA methylation, miRNA expression in the epidermis appears to be highly responsive to UV exposure, both acute and chronic. For example, an early study noted that acute UV exposure caused changes in expression of 44 miRNAs, occurring in a time-dependent manner, in human keratinocytes [72] while chronic skin exposure to sun caused changes in expression of 55 miRNAs of which three, miR-383, miR-34a, and miR-134 were also upregulated during chronological aging [68]. On the whole, chronic skin exposure gives rise to differences in mRNA expression that largely exceed those occurring during aging and, moreover, the type and direction of changes in miRNAs are different in infants and aged individuals [68]. Studies on keratinocytes and other cells proved that miRNAs mobilization serves mainly to protect cells from UV-induced DNA damage and its propagation through cell proliferation [73].

Wound healing is a relatively rapid, dynamic process triggered to restore the damaged epidermal barrier. After initial wound clotting by platelets and immune cell mobilization (inflammatory phase), dermal fibroblasts start to fill the wound (granulation phase), which is then covered by keratinocytes migrating from the wound edge (re-epithelization phase). Shaw and Martin [74] have shown that expression of *Ezh1* and *Ezh2*, encoding H3K27 methyltransferases was transiently downregulated, and the level of the repressive H3K27me3 mark was substantially reduced in keratinocytes during wound healing while Jmjd3, the respective demethylase, was upregulated. These changes were associated with the de-repressed transcription of a large set of “repair genes.” Jmjd3 was specifically upregulated at the wound edge and found, along with erasure of the H3K27me3 histone mark, on promoters of genes encoding multiple interleukins, matrix metalloproteinases, and growth factors activated during the inflammatory phase [75]. It was also found that the activation of histone acetyltransferases or inhibition of deacetylases promoted wound closure [76]. Accordingly, changes in the acetylation status of histone H4 lysine residues were observed at the wound margin in mice [77].

There is a lot of studies concerning the role of miRNAs in the wound healing process. For example, thermal injury to epidermal stem cells was shown to alter the expression of 33 miRNAs, most of which, based on bioinformatics analysis, regulated key processes involved in wound healing, such as cell proliferation and differentiation, cell growth, apoptosis, cell adhesion, and migration [78]. As many as 54 miRNAs were found to be differentially expressed during the granulation stage of wound healing [79] and 200 miRNAs throughout the entire time course of this process [80]. Among the most highly upregulated miRNAs was miR-21, known to stimulate keratinocyte proliferation and migration [81]. Interestingly, while histone modifications and DNA methylation represent an “intrinsic” mechanism of keratinocyte reaction to wounding, miRNAs can also be produced by other cell types, e.g., dermal fibroblasts or epidermal immune cells, and transported via extracellular vesicles to regulate keratinocyte proliferation and migration [82].

## 5. Conclusions

Accumulated data clearly demonstrate that epigenetic mechanisms are involved in the regulation of multiple aspects of epidermal growth and differentiation. In particular, they exert control over the balance between stem cell quiescence and proliferation, which is essential for constant epidermal renewal and becomes critical during wound re-epithelialization. Epigenetic factors also control the onset and proper pace of keratinocyte differentiation. Furthermore, epigenetic mechanisms are activated in response to acute external stress, upon which their action seems to protect epidermal integrity and function and may also help epidermal cells to adjust to long-term physiological changes. As shown by numerous studies concerning skin pathologies, changes in the pattern of epigenetic marks in the epidermis precede the clinical symptoms and thus may aid in the early diagnosis of skin diseases. Even more important are results showing that drugs, which reverse or attenuate changes in epigenetic marks, also attenuate the disease’s symptoms. We are still far from comprehending the extent and the complexity of the contribution of epigenetics to epidermal homeostasis, and further research efforts should be undertaken in this field. Possibly, with the advantage of high-throughput, quantitative technologies, such as ChIP-seq or RNA-seq, novel epigenetic factors essential for proper epidermal differentiation and those critically disturbed in multiple skin pathologies will be identified.

## Data Availability

Data is contained within the article or supplementary material.

## Supplementary Materials

The following are available online at https://www.mdpi.com/2075-4655/5/1/1/s1, Table S1: Effect of knockout/down, overexpression or mutation of histone modifying enzymes on keratinocyte/epidermal growth and differentiation.
