# Supplementary material for: Epigenetic Regulation of Epidermal Differentiation
Source: Epigenomes. 2021 Jan 1;5(1):1. doi: 10.3390/epigenomes5010001 (PMC8594726; doi:10.3390/epigenomes5010001)
Supplement: Supplementary file 1 [file epigenomes-05-00001-s001.pdf]

Tissue/cell	Modified gene	Altered histone level	Effect on keratinocyte/epidermal growth and differentiation	Ref.
Mouse epidermis	**Setd8** knockout[1]	H3K20me1 ↓	Inhibition of progenitor cell proliferation; Impaired differentiation	[10]
Mouse epidermis	**Ezh2** knockout[1]	H3K27me3 ↓	Inhibition of proliferation; Premature differentiation	[15]
Mouse epidermis;	**Hdac1/Hdac2** knockout [1]	acH3 ↓	Enhanced proliferation; epidermal hyperplasia; disturbed hair follicle differentiation	[19]
Primary human keratinocytes	**Jmjd3** knockdown	H3K27me3 ↑	Blocked differentiation	[16]
	**Jmjd3** overexpression	H3K27me3 ↓	Enhanced expression of differentiation markers	
Dog nasal epidermis	**Suv39H2** Missense mutation (inactive enzyme)	H3K9me3 ↓	Aberrant terminal differentiation	[18]
HaCaT cells	**Suv39H1** knockout	H3K9me3 ↓	Increased expression of some differentiation genes	[17]
HaCaT cells; reconstituted epidermis	**Jarid1b** knockdown	H3K4me3 ↑	Delayed differentiation	[20]
	**Jarid1b** overexpression	H3K4me3 ↓	Reduced proliferation; Enhanced differentiation	
Mouse epidermis	**Ash1l** Mutation (reduced expression)	H3K36me3 ↓	Hyperproliferation; Disturbed differentiation	[21]

Table S1. Effect of knockout/down, overexpression or mutation of histone modifying enzymes on keratinocyte/epidermal growth and differentiation

[1] – conditional knockout in the basal epidermal layer (Cre recombinase expressed under keratin 14 gene promoter); ↓- decrease in modified histone level; ↑- increase in modified histone level.
